# Validation of Two Portable Instruments to Measure Iron Concentration in Groundwater in Rural Bangladesh

**DOI:** 10.3329/jhpn.v27i3.3384

**Published:** 2009-06

**Authors:** Rebecca D. Merrill, Abu Ahmed Shamim, Alain B. Labrique, Hasmot Ali, Kerry Schulze, Mahbubur Rashid, Parul Christian, Keith P. West, Jr.

**Affiliations:** ^1^ Department of International Health, Johns Hopkins Bloomberg School of Public Health, 615 North Wolfe Street, Baltimore, MD 21205, USA; ^2^ JiVitA Project, House 63, Road 3, Karanipara, Rangpur, Bangladesh

**Keywords:** Groundwater, HACH, Iron, Test-kits, Tubewell, Water analysis, Bangladesh

## Abstract

Iron is ubiquitous in natural water sources used around the world for drinking and cooking. The health impact of chronic exposure to iron through water, which in groundwater sources can reach well above the World Health Organization's defined aesthetic limit of 0.3 mg/L, is not currently understood. To quantify the impact of consumption of iron in groundwater on nutritional status, it is important to accurately assess naturally-occurring exposure levels among populations. In this study, the validity of iron quantification in water was evaluated using two portable instruments: the HACH DR/890 portable colorimeter (colorimeter) and HACH Iron test-kit, Model IR-18B (test-kit), by comparing field-based iron estimates for 25 tubewells located in northwestern Bangladesh with gold standard atomic absorption spectrophotometry analysis. Results of the study suggest that the HACH test-kit delivers more accurate point-of-use results across a wide range of iron concentrations under challenging field conditions.

## INTRODUCTION

Iron, the second most abundant metal on earth, is ubiquitous in water resulting from the geological formations over and through which water flows, the pH and temperature of the water, and the concentration of oxygen that comes in contact with the water (1,2). Iron concentration of treated water is often below the official treatment goal of 0.3 mg/L, a cut-off defined by the World Health Organization (WHO) and used by international agencies based on changes to aesthetic qualities of water above this level (primarily in taste and smell) (2,3). Surface water also commonly has low levels of iron as a result of the oxidation of soluble ferrous (Fe^2+^) to insoluble ferric (Fe^3+^) iron (4). Individuals consuming untreated groundwater, depending on geographic location, may be routinely consuming water with iron concentrations as high as 200 times the aesthetic limit of WHO (5).

A first step towards mapping the exposure to iron in groundwater and evaluating its potential nutritional contribution to populations is to have portable field instruments that can accurately measure iron content in water. Several instruments are now commercially available. This study was carried out to compare the performance of two widely-marketed instruments: the HACH DR/890 portable colorimeter (Hach Company, Loveland, CO, USA, hereafter referred to as ‘colorimeter') and HACH iron test-kit, model IR-18B (Colour disc, 0.2-10 mg/L), hereafter referred to as ‘test-kit' against the gold standard atomic absorption spectrophotometry (AAS).

## MATERIALS AND METHODS

Twenty-five tubewells located over an approximate rural area of 6.5 sq km in Gaibandha district of northwest Bangladesh were included in this study. Characteristics of tubewells were recorded, including the material surrounding the base and the ‘presence of arsenic' based on the colour of the tubewell-mouth at the time of sampling (many grassroots-level NGOs in Bangladesh have been testing tubewells and painting those red with arsenic concentrations equal to or above 50 µg/L and green otherwise) or responses from local residents who use the well.

Before analysis of water, wells were pumped for five minutes to avoid stagnation of sampling water in the tubewell piping. Water temperature to the nearest 0.1 °C (portable thermometer, model no. ST-9269A/B/C, Winning Technology Ltd., Hong Kong) and pH to the nearest 0.1 (ADWA AD100 pH Electronic Meter, Adwa Instruments, Belgium) were recorded. Total iron concentration was initially determined to the nearest 0.1 mg/L by the test-kit. Analysis began with no dilution and, if the result exceeded the 10.0 mg/L detection limit, proceeded sequentially with 2- and 5-fold dilutions. If the limit was exceeded when using the 5-fold dilutions, total iron concentration was recorded as >50.0 mg/L. Next, total iron concentration was analyzed with the colorimeter, to the nearest 0.01 mg/L, using the FerroVer method. Analysis began with no dilution and, if the 3.30 mg/L limit was exceeded, was repeated sequentially with 2-, 5-, 10-, and 20-fold dilutions. If the limit was exceeded when using the 20-fold dilution, total iron concentration was recorded as >66.00 mg/L. Finally, ferrous iron concentration, to the nearest 0.01 mg/L, was measured by the colorimeter using 1,10 Phenanthroline at dilution factors of 1, 2, 5, 10, and 25, as required based on a detection limit of 3.30 mg/L. Trained personnel carried out all analyses on-site, routinely observed by a supervisor, who collected a fresh groundwater sample for each test.

A water sample from each selected tubewell was collected in a trace element-free PET bottle and immediately acid-preserved to a pH of <2 using HCl. Each day, the acid-preserved water samples were transported to a local project office in Gaibandha where they were stored below 20 °C in a dark room. Within one week of collection, all water samples were measured for total iron by hydride generation–AAS (Varian SpectrAA 220, Varian, Inc., Palo Alto, CA, USA) at the Bangladesh Council of Scientific and Industrial Research (BCSIR), Dhaka.

Descriptive information was compared using the Kruskal-Wallis test, accounting for ties. Correlations between covariates and iron concentration were determined using non-parametric methods of analysis (Spearman's rank correlation) due to the skewed distribution of iron concentration in this sample. The differences between the results of total iron concentration for each instrument were explored using non-parametric (Wilcoxon- signed rank and Kruskal-Wallis tests) analysis and Bland Altman methods (6). Linear and curvilinear regression methods were used for modelling the differences in results between instruments. All data analyses were performed using the Stata software (version 9.2) (StataCorp, College Station, TX) (7).

## RESULTS

**Fig. 1.**
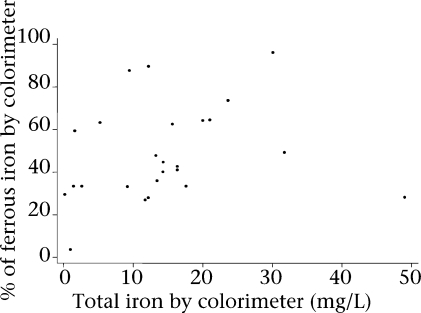
Ferrous iron as a percentage of total iron defined by the colorimeter [percent ferrous iron=435.6+total iron (mg/L)]∗(−0.003, p>0.99)+pH∗(5.5, p=0.73)+temperature (°C)∗(-16.0, p=0.11)

Tubewell structure and water characteristics are presented in Table 1. Total iron concentration (mg/L), defined by AAS, did not differ by material surrounding the base, tubewell colour, sediment in the water, or reported presence of arsenic (p>0.10 by the Kruskal-Wallis test). Tubewell colour was not associated with the presence of arsenic as reported by local residents (p=0.37 by Spearman rank correlation) (Table 2). Total iron concentration, defined by AAS, negatively correlated with temperature (r= −0.33) and positively correlated with pH (r=0.46). Ferrous iron (mg/L), defined by the colorimeter, showed similar correlations with temperature (r= −0.35) and pH (r=0.49) but, as a percentage of total iron, was not associated with either (p>0.45) (Fig. 1).

**Table 1.** Characteristics of tubewells (n=25) in rural Gaibandha district, Bangladesh

Characteristics	Value	
	No.	%
Material surrounding the base		
Mud	10	40
Brick	2	8
Small amount of cement	2	8
Large cement platform	11	44
Tubewell colour		
Natural	21	84
Green	3	12
Red	1	4
Sediment in water		
Iron	0	0
Sand	1	4
Iron and sand	3	13
None	21	84
Arsenic present∗		
No (<50 µg/L)	2	8
Yes (≥50 µg/L)	2	8
Never tested	21	84
	Mean (SD) and range	
Temperature (°C)	26.8 (0.5)	
	25.8-28.6	
pH	7.3 (0.3)	
	6.5-7.8	
	Median (IQR) and range	
Total iron concentration (mg/L)		
AAS	11.4 (8.4-15.8)	
	0.2-28.0	
Test-kit	12.8 (10.8-18.8)	
	0.6-32.0	
Colorimeter	13.60 (9.60-17.60)	
	0.50-49.20	
Ferrous iron by colorimeter (mg/L)	6.32 (3.10-10.50)	
	0.02-28.60	

∗Based on asking local users ‘Does this tubewell contain arsenic?', reflecting results from arsenic-awareness campaigns offering free field-testing 4-6 years prior to testing of the two devices in the present study

AAS=Atomic absorption spectrometry; IQR=Interquartile range; SD=Standard deviation

**Table 2.** Relationship between tubewell colour and reported arsenic level

Presence of arsenic∗	Tubewell colour		
	Natural	Green	Red
No (<50 µg/L)	2	0	0
Yes (≥50 µg/L)	2	0	0
Never tested	17	3	1

∗Based on asking local users ‘Does this tubewell contain arsenic?', reflecting result from arsenic-awareness campaigns offering free field-testing 4-6 years prior to testing of the two devices in the present study

**Fig. 2.**
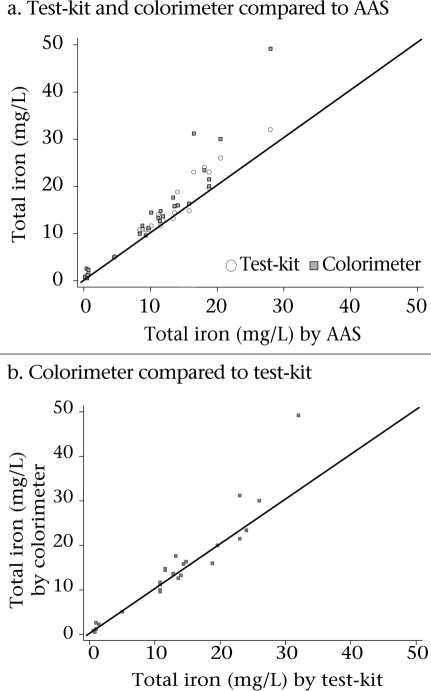
Scatter plots comparing total iron concentration (mg/L) results from AAS, colorimeter, and test-kit (line of equality included)

The colorimeter and test-kit results were significantly higher than AAS (median difference (mg/L) (IQR): colorimeter–AAS, 1.85 (1.20-3.25) and test-kit–AAS, 1.10 (0.40-2.8), p<0.001 based on Wilcoxon-signed rank test) but were not significantly different from each other (median difference (mg/L) (IQR): 0.39 (-0.60–1.59), p>0.05 Wilcoxon signed rank test) (Fig. 2). The Spearman rank correlation for pair-wise comparisons of all three instruments ranged from 0.96 between the colorimeter and the test-kit to 0.98 between the test-kit and AAS.

AAS=Atomic absorption spectrophotometer

**Fig. 3.**
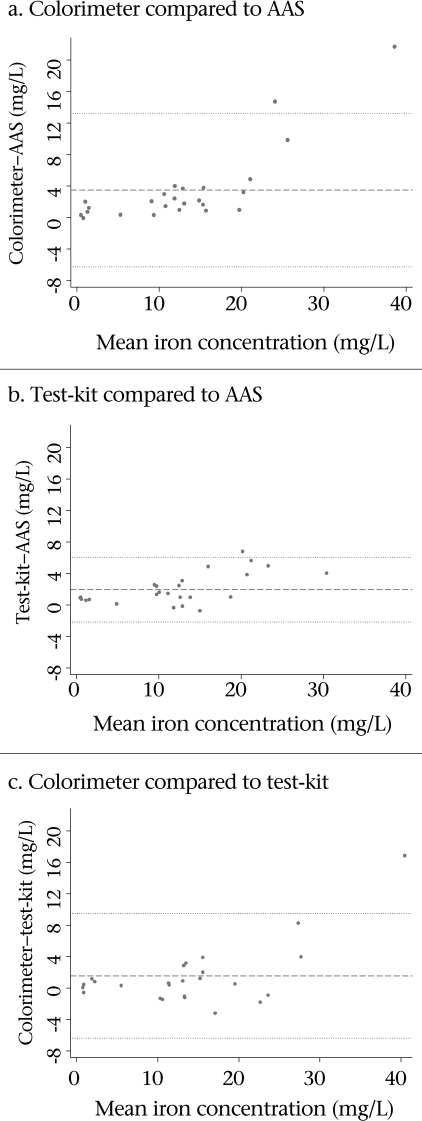
Difference against mean total iron concentration (mg/L) comparing colorimeter, t est-kit, and AAS results (Dashed line: mean difference, dotted lines: 2∗SD of mea n difference)

**Fig. 4.**
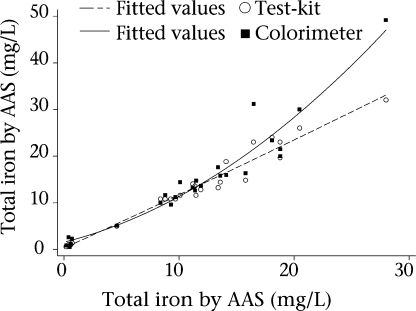
Curvilinear models of total iron results by field instrument against AAS

For both colorimeter and test-kit, the difference between the field instrument and the AAS results significantly increased, after controlling for pH and temperature, as the mean total iron concentration of the two measurements increased (Fig. 3). When comparing the colorimeter and AAS results, the magnitude of the difference significantly increased with iron concentration above 15 mg/L (p<0.001 for the linear spline term representing iron concentration ≥15 mg/L). The difference between the test-kit and AAS showed a small but significant increase when the mean fell below 20 mg/L (p<0.02 for the linear spline term representing iron concentration <20 mg/L). Curvilinear analysis was carried out to model the relationship between the results for each instrument and AAS controlling for pH and temperature (Fig. 4).

AAS=Atomic absorption spectrophotometer; SD=Standard deviation

AAS=Atomic absorption spectrophotometer

## DISCUSSION

This study was conducted to investigate the accuracy of two commercially-available field-test instruments: the HACH DR/890 portable colorimeter and HACH iron test-kit, Model IR-18B, designed to measure total (and ferrous) iron concentration in groundwater. The experiment was carried out by sampling water from 25 rural tubewells in northwest Bangladesh. Total iron concentration estimates from field analysis by both the portable devices were compared with each other and with values obtained from AAS, considered a gold standard.

Descriptive information about the tubewell, including the presence of arsenic as reported by local residents or observing whether the tubewell was painted red or green, did not help predict iron concentration. Other attributes of water, including temperature and pH, negatively and positively correlated, respectively, to total and ferrous iron concentration, as expected (1,2,5) and were used to help predict the total iron concentration of water samples.

Total iron concentration results measured by colorimeter and test-kit highly correlated with the gold standard AAS results (≥0.96); however, there were significant differences in absolute results. Notably, as total iron concentration increased to above 15 mg/L, the colorimeter results began to diverge more rapidly from the AAS results compared to results below 15 mg/L, even after removing the most extreme colorimeter test value (49.2 mg/L). The colorimeter's loss of accuracy raises concern about its utility in geographic areas such as in northern Bangladesh where iron concentrations in tubewell water can reach above 30 mg/L (5).

Field conditions may have contributed to the differences among the test-kit, colorimeter and AAS results. While the presence of interfering factors, such as calcium when present at concentrations of more than 10,000 mg/L or magnesium when above 100,000 mg/L, or extreme pH, are rarely encountered in this region (5), organic matter and colloidal bodies present in groundwater will chelate iron and may affect the amount of soluble iron available at the time of field analysis and after acid preservation (8). Instrument-specific protocol may account for some variation. The test-kit requires subjective analysis and natural light, which is affected by the weather, to determine an exact match between the sample colour and a shade of red on a colour wheel. Consistent colorimeter readings require that the sample is placed tightly in the instrument and that there is no machine movement during analysis. Under conditions of high iron content in groundwater and difficult field conditions, such as those encountered in northern rural Bangladesh, our findings suggest that the HACH test-kit is superior to the HACH colorimeter in the field estimation of total iron concentration in tubewell water.

## ACKNOWLEDGEMENTS

The study was conducted under the auspices of the United States Agency for International Development (USAID)/Bangladesh Health and Population Programmes (UBHPP) of the Ministry of Health and Family Welfare (MoHFW), Government of Bangladesh (GoB). JiVitA Project research activities are made possible through the generous support of the Office of Health, Infectious Diseases and Nutrition (Grant No. GHS-A-00-03-00019-00), USAID (Washington DC, USA), the USAID Mission in Dhaka, the Bill & Melinda Gates Foundation (Seattle, WA, USA) and the GoB, with additional financial and technical assistance from SIGHT AND LIFE and the SIGHT AND LIFE Research Institute, Johns Hopkins University, Baltimore, MD, USA.

The authors also thank Dr. Shofiul Alam for helping manage the study and the study technicians for their diligent field work.
